# Identification and validation of a ferroptosis-related gene signature predictive of prognosis in breast cancer

**DOI:** 10.18632/aging.203472

**Published:** 2021-09-09

**Authors:** Qin Liu, Jian-Ying Ma, Gaosong Wu

**Affiliations:** 1 Department of Thyroid and Breast Surgery, Zhongnan Hospital of Wuhan University, Wuhan, China; 2 Department of Breast Surgery, Thyroid Surgery, Huangshi Central Hospital of Edong Healthcare Group, Hubei Polytechnic University, Huangshi, Hubei, China

**Keywords:** breast cancer, ferroptosis, recurrence-free survival, immune cell infiltration, tumor microenvironment

## Abstract

Ferroptosis, a novel form of regulated cell death, is closely associated with the occurrence and development of malignant tumors. Here, we utilized a bioinformatics approach to identify ferroptosis-related genes to establish a robust and reliable prognostic signature in breast cancer (BC). Univariate Cox regression and LASSO regression analyses of patient’s survival and gene expression data identified a prognostic signature consisting of 10 ferroptosis-related genes (FRGs). The signature demonstrated a favorable prediction performance, and was validated in two independent datasets, GSE21653 and GSE25066. Analyses of immune infiltrates, tumor microenvironment, immune checkpoints, mutations, drug sensitivity, and clinicopathological features revealed significant differences between low- and high-risk BC patients. A multivariate analysis revealed that the signature was an independent prognostic predictor in BC, and a nomogram combining the risk score and tumor stage intuitively displayed high accuracy and reliability with respect to predicting the survival outcomes of BC patients. These findings indicate that the identified prognostic signature is a potential indicator predictive of prognosis and immunotherapeutic responses in BC patients.

## INTRODUCTION

Breast cancer (BC) is the most common cancer in women and the leading cause of cancer death and disability-adjusted life years among females worldwide [1, 2]. A study based on the 2017 Global Burden of Disease (GBD) conducted in 195 countries and regions from 1990 to 2017 showed that the global incidence of BC increased to 1,960,681 cases in 2017, and the global incident cases of BC increased by 123% between 1990 and 2017 [2]. Recently, according to the World Health Organization, there were 2.3 million new BC cases in 2020, thereby replacing lung cancer as the most common cancer [3]. With 1 in 6 cancer mortality, BC is the leading cause of cancer death [4]. BC is a highly heterogeneous malignant tumor composed of many subtypes that differ in biological behavior, clinical outcomes, and therapeutic responses [5]. The diversity between and within tumors as well as among individuals all together determine the prognosis and drug resistance of BC [6].

Ferroptosis is a novel form of programmed cell death, which is defined as an oxidized, iron-dependent form of regulatory cell death [7]. Ferroptosis has unique morphological and biochemical characteristics, including mitochondrial contraction, mitochondrial membrane rupture, increase in lipid reactive oxygen species, consumption of glutathione, and loss of glutathione peroxidase 4 [8, 9]. Ferroptosis participates in carcinogenesis and cancer development in various cancers [10]. Ferroptosis takes part in maintaining the survival of normal cells and tissue, some highly aggressive malignancies also have been identified as intrinsically susceptible to ferroptosis [11]. Multiple genes, including P53 [12], ACSL4 [13], SLC7A11 [14], and FZD7 [15] modulate sensitivity to ferroptosis and can serve as markers of ferroptosis. Ferroptosis has been recently suggested as a promising target to inhibit tumor growth and trigger cell death, especially in malignant tumors that are resistant to traditional therapies [16]. Roles and molecular mechanisms of ferroptosis in BC have been investigated in several studies [17, 18]. Recent studies have investigated the key mechanisms regulating ferroptosis in BC [19]. However, the expression patterns and the prognostic values of ferroptosis-related genes in BC are still largely unknown. Moreover, prognostic models integrating multiple biomarkers help clinicians make treatment decisions and develop optimal treatment combinations to reduce disease mortality.

In this study, we utilized a bioinformatic approach to identify the ferroptosis-related genes (FRGs) to establish a robust, reliable prognostic signature in BC. This signature was validated by two independent sets and demonstrated a favorable prediction performance. Analysis of immune infiltrates, tumor microenvironment (TME), immune checkpoints, mutations, drug susceptibility, and clinicopathological features revealed significant differences between low- and high-risk BC patients. Our data indicate that this FRGs prognostic signature may improve prognosis predictions and immunotherapy responses in BC patients.

## RESULTS

### Identification of FRGs in BC patients

**Figure 1:**
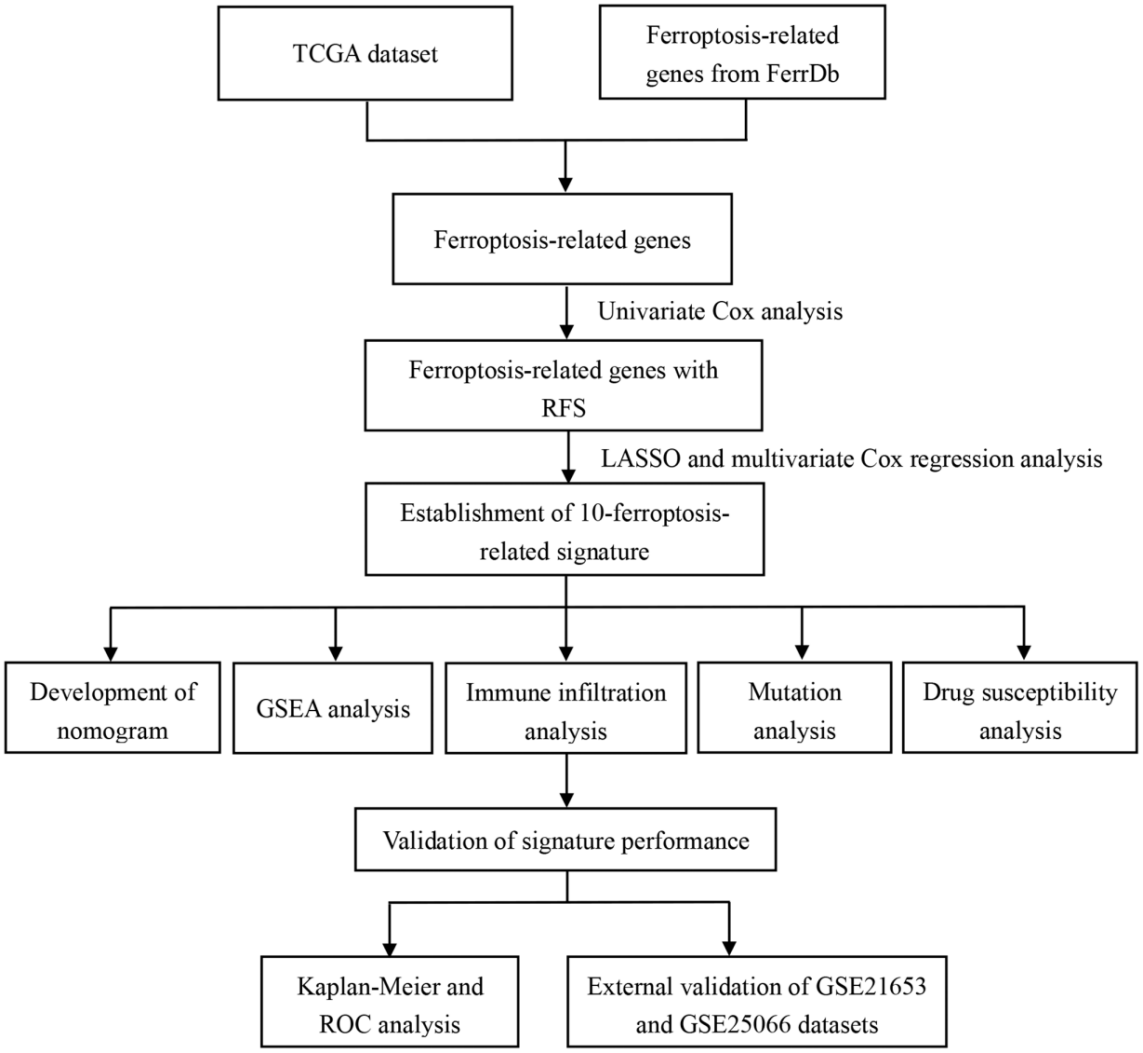
The flow chart of data analysis in this study.

Figure 1 shows the research idea of the present study. The detailed clinicopathological features of BC patients are summarized in Table 1. 267 FRGs were acquired from the FerrDb database and integrated with the mRNA data from TCGA database to obtain 255 FRGs. Through merging the candidate genes with two GEO external validation sets, 240 common FRGs were obtained for modeling analysis.

**Table 1:** Clinicopathologic characteristics of breast cancer patients included in this study.

**Variables**	**TCGA cohort**	**GSE21653 dataset**	**GSE25066 dataset**
	**Number (%)**	**Number (%)**	**Number (%)**
Age			
< 60	172 (54.1)	135 (56.0)	244 (79.0)
≥60	146 (45.9)	106 (44.0)	65 (21.0)
Surgery type			
Lumpectomy	109 (34.3)	/	/
Modified Radical Mastectomy	109 (34.0)	/	/
Other	101 (31.8)	/	/
Margin status			
Negative	291 (91.5)	/	/
Positive	16 (5.0)	/	/
Unknow	11 (3.5)		/
Tumor status			
Tumor free	256 (80.5)	/	/
With tumor	40 (12.6)	/	/
Unknow	22 (6.9)		/
T stage			
T1	93 (29.2)	55 (22.8)	22 (7.1)
T2	174 (54.7)	121 (50.2)	165 (53.4)
T3–4	51 (16.0)	65 (27.0)	122 (39.5)
N stage			
N0	149 (46.9)	113 (46.9)	87 (28.2)
N1–3	169 (53.1)	128 (53.1)	222 (71.8)
Stage			/
I	54 (17.0)	/	6 (1.9)
II	175 (55.0)	/	165 (53.4)
III–IV	79 (24.8)	/	134 (43.4)
Unknow	10 (3.1)	/	4 (1.3)

### Establishment of ferroptosis-related gene signature

**Figure 2:**
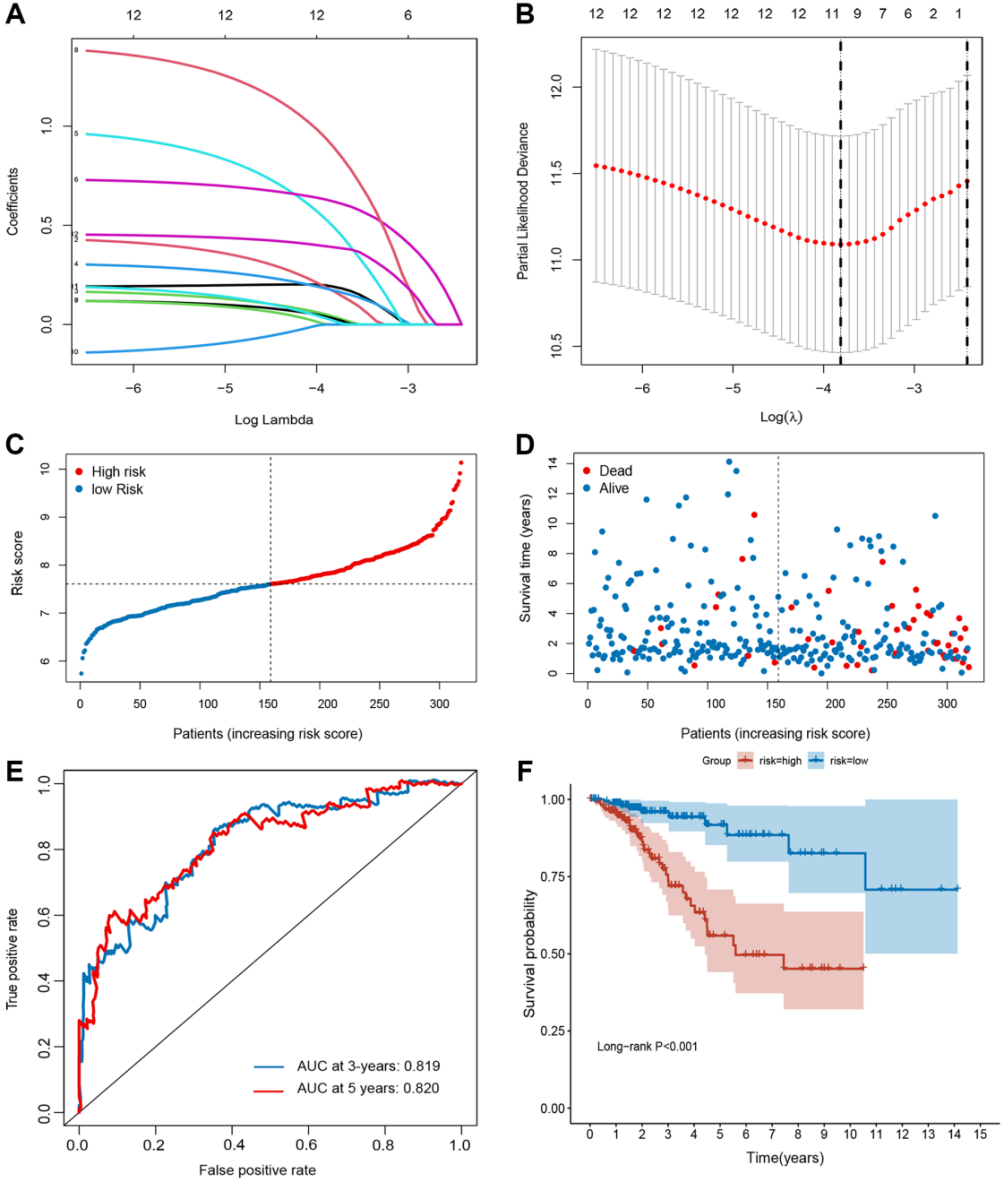
**Establishment of ferroptosis-related gene signature in TCGA set.** (**A** and **B**) The LASSO regression analysis and partial likelihood deviance of the 12 prognosis-associated FRGs. (**C**) The ranked dot plot indicates the risk score distribution. (**D**) Scatter plot illustrates the patients’ survival status. (**E**) ROC curve of 10-FRG signature. (**F**) Kaplan-Meier method was used to plot the RFS curve for the high-risk and low-risk score groups.

Univariate Cox regression based on survival and gene expression data of BC patients was used to evaluate the prognostic role of the FRGs. In total, 12 FRGs were selected as candidate genes to construct the prognostic model with the criterion of *P* value < 0.05 (Supplementary Figure 1). LASSO regression analysis was performed using the training set to identify genes that exhibited the highest association with RFS (Figure 2A and 2B). Finally, 10 genes (CHAC1 [OMIM 614587], GCLM [OMIM 601176], SLC7A11 [OMIM 607933], HMOX1 [OMIM 141250], NOX4 [OMIM 605261], DUOX1 [OMIM 606758], TFR2 [OMIM 604720], WIPI2 [OMIM 609225], DRD4 [OMIM 126452], and NGB [OMIM 605304]) were selected to build the prognostic signature in the training set. Risk score = (0.19003 × ExpCHAC1) + (0.16688 × ExpGCLM) + (0.03959 × ExpSLC7A11) + (0.17125 × ExpHMOX1) + (0.49082 × ExpNOX4) + (0.61939 × ExpDUOX1) + (0.02989 × ExpTFR2) + (0.89319 × ExpWIPI2) + (0.01862 × ExpDRD4) + (0.38955 × ExpNGB). The cut-off value for the low- and high-risk groups was 0.936, which was calculated by the “survminer” R package. The distributions of RFS status were shown in Figure 2C and 2D. The AUCs were 0.819 and 0.820 for the 3- and 5-year RFS rates in the training set, suggesting a great prognostic value of this signature (Figure 2E). Moreover, our data demonstrated that the mortality rate in the low-risk group was markedly lower than that in the high-risk group (Figure 2F).

### Validation of the ferroptosis-related signature

**Figure 3:**
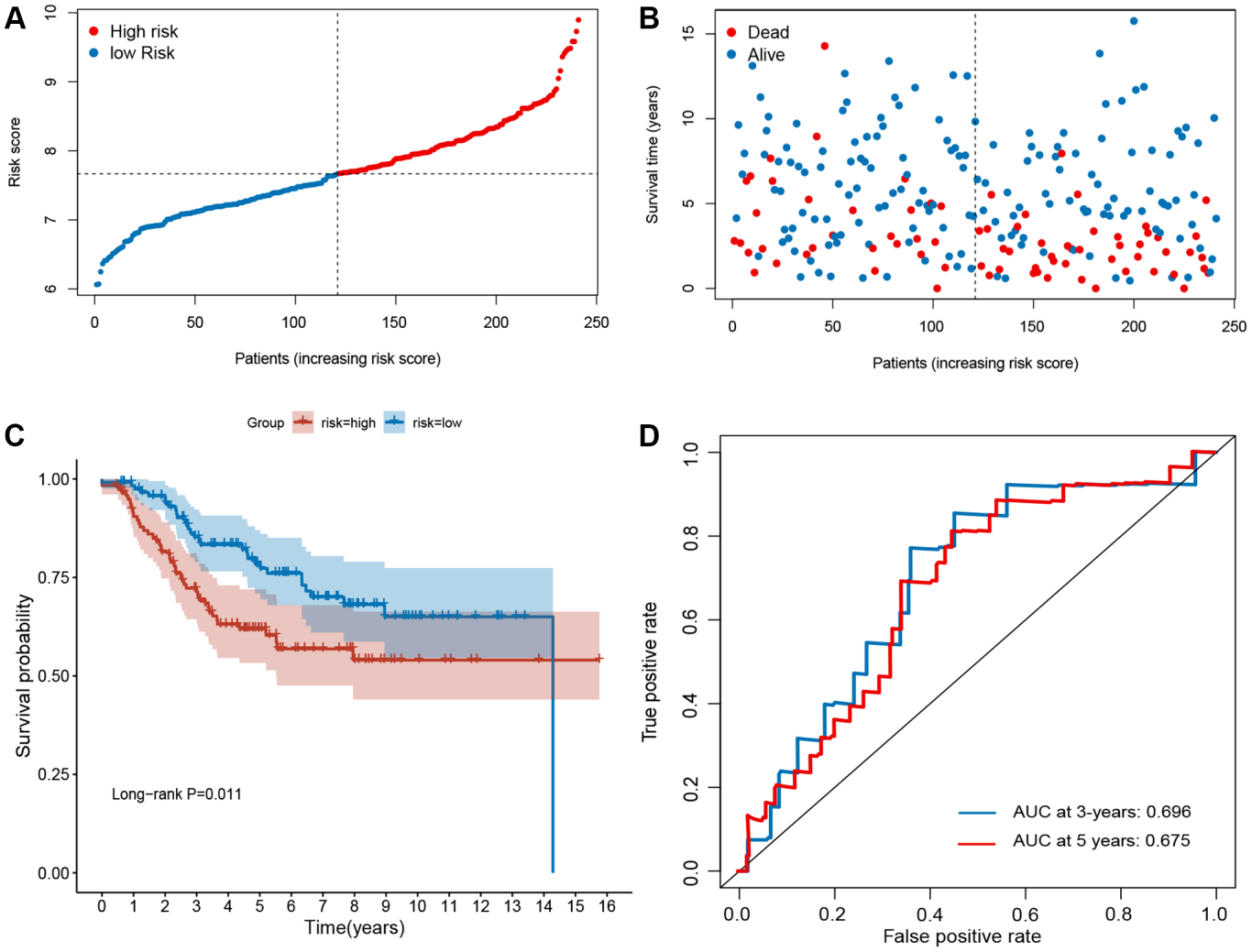
**Evaluation of ferroptosis-related gene signature in GSE21653 dataset.** (**A**) The ranked dot plot indicates the risk score distribution. (**B**) Scatter plot illustrating the patients’ survival status. (**C**) Kaplan-Meier method was used to plot the RFS curve for the high-risk and low-risk score groups. (**D**) ROC curve of the 10-FRG signature.

**Figure 4:**
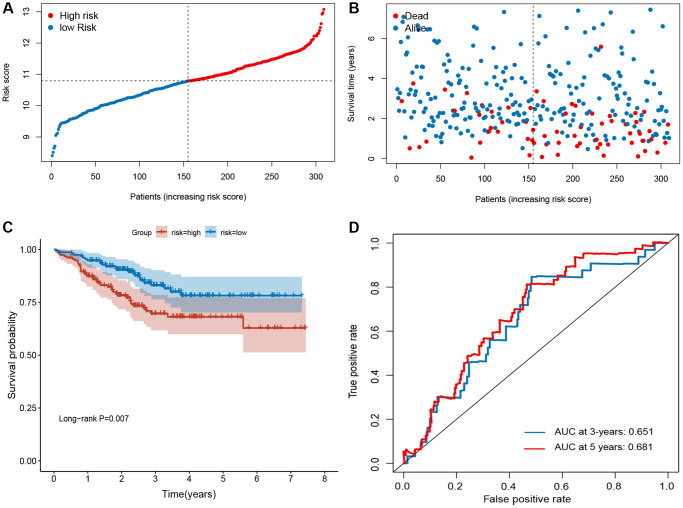
**Evaluation of ferroptosis-related gene signature in GSE25066 dataset.** (**A**) The ranked dot plot indicates the risk score distribution. (**B**) Scatter plot illustrates the patients’ survival status. (**C**) Kaplan-Meier method was used to plot the RFS curve for the high-risk score and low-risk score groups. (**D**) ROC curve of 10-FRG signature.

To validate the robustness of the signature, the validation sets from GEO (GSE21653 and GSE25066) were also stratified into high- or low-risk groups based on the same formula as that from the training set (Figures 3 and 4). The distributions of RFS status were shown in Figure 3A–3B and 4A–4B. The patients with high-risk score had a poor RFS than the low-risk patients (Figures 3C and 4C). The AUC of ROC for 3- and 5-year survival predictions were 0.696 and 0.675 in the GSE21653 dataset and 0.651 and 0.681 in the GSE25066 dataset. These data indicate that the model has a favorable performance (Figures 3D and 4D).

### Immune infiltration analysis

**Figure 5:**
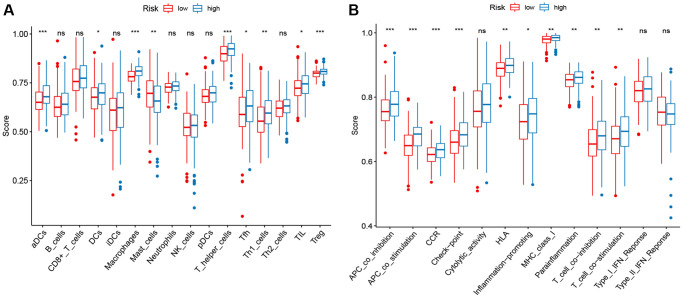
**ssGSEA scores in high-risk and low-risk patients in the TCGA set.** The scores of 16 immune cells (**A**) and 13 immune-related functions (**B**) are displayed in boxplots.

**Figure 6:**
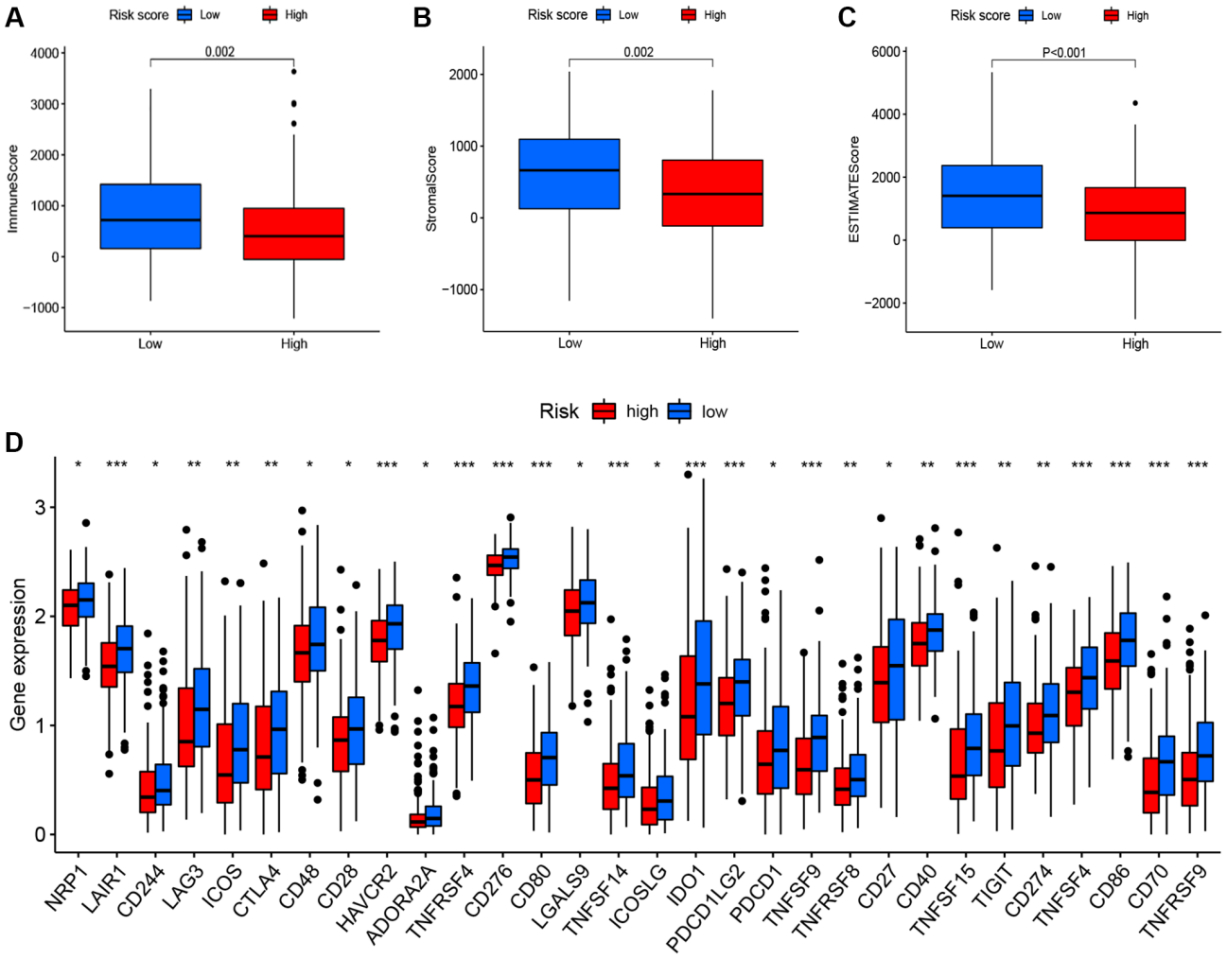
**Correlation between the risk score, the TME score, and immune checkpoints.** (**A**) immune scores. (**B**) stromal scores. (**C**) ESTIMATE scores. (**D**) immune checkpoints.

To determine whether different risk-stratified patients were characterized by different immune infiltrates and TME, we compared the immune infiltrates and TME in high- and low-risk patients. As shown in Figure 5A, aDCs, DCs, macrophages, Treg cells, T helper cells, Tfh, Th1 cells, and TIL were highly infiltrated in the low-risk group (*P* < 0.05). Furthermore, significant differences in most immune-related pathways were observed between the two risk groups (*P* < 0.05; Figure 5B). Analysis of the relationship between the risk score and the TME score revealed that a significant negative association between the risk score and stromal score, immune score, and ESTIMATE score (Figure 6A–6C). In addition, we found that the expression of 4 immune checkpoints, including PD-1 (PDCD1), PD-L1 (CD274), and CTLA-4 was significantly upregulated in the low-risk group (Figure 6D), suggesting a potential role of the signature model in predicting immune responses to immunotherapy in BC patients.

### Comprehensive analysis of the signature

**Figure 7:**
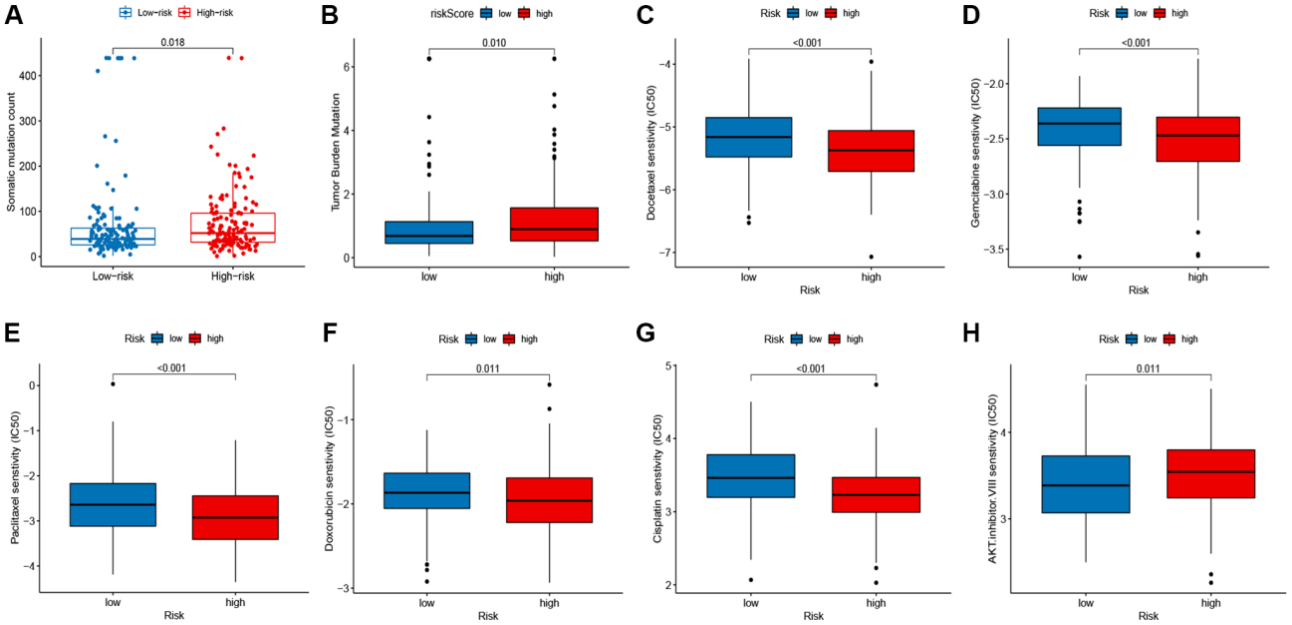
**Correlation between the signature, mutation status, and drug susceptibilities.** (**A**) somatic mutation count in the high- and low-risk groups. (**B**) tumor mutation burden (TMB) in the high- and low-risk groups. (**C**–**H**) drug susceptibilities in the high- and low-risk groups.

Next, we assessed the relationship of the signature with the somatic mutation count and tumor mutation burden (TMB). The results indicated that the group of high risk owned a higher somatic mutation count (*P* = 0.018; Figure 7A) and TMB (*P* = 0.01; Figure 7B) than the group of low risk. In addition, we analyzed the responses of patients with different risk groups to antineoplastic drugs. As shown in Figure 7C–7H, we observed significant differences in the IC50 values of 6 antineoplastic drugs between the two risk groups. Patients with high-risk were more sensitive to Gemcitabine (*P* < 0.001), Paclitaxel (*P* < 0.001), Doxorubicin (*P* = 0.011), Docetaxel (*P* < 0.001), and Cisplatin (*P* < 0.001), while patients with low-risk were more sensitive to AKT inhibitor VIII (*P* < 0.001).

### Association between signature and clinicopathological features

**Figure 8:**
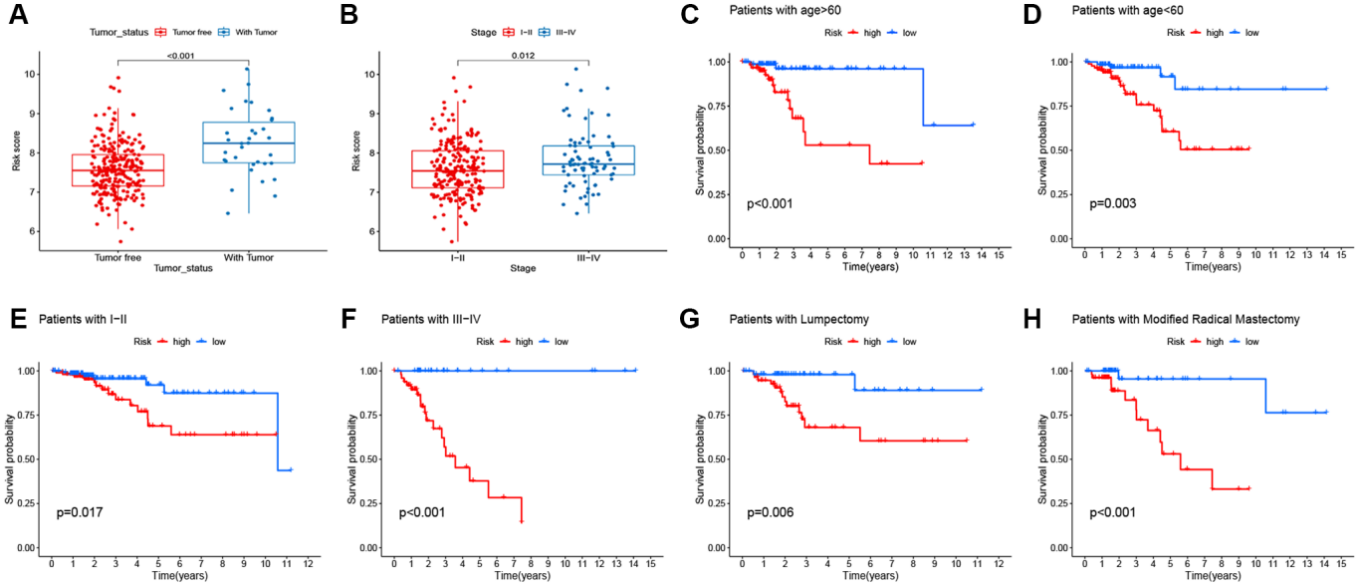
**Correlation between signature and clinicopathological features.** (**A**–**B**) Correlation of signature with clinicopathological features. (**C**–**H**) the predictive performance of the signature in different subgroups.

The correlation between the prognostic signature and clinical features was analyzed. The boxplot demonstrated that the signature was significantly linked to tumor status (*P* < 0.001; Figure 8A) and tumor stage (*P* = 0.012; Figure 8B). In addition, stratification analyses were conducted to examine whether the signature retained the ability to predict RFS in various subgroups. Compared with low-risk patients, BC high-risk patients had a worse RFS in subgroups stratified by age (< 60 vs. ≥ 60; Figure 8C–8D), stage (I–II vs. III–IV; Figure 8E–8F), and surgery type (lumpectomy vs. modified radical mastectomy; Figure 8G–8H).

### Building a predictive nomogram

**Figure 9:**
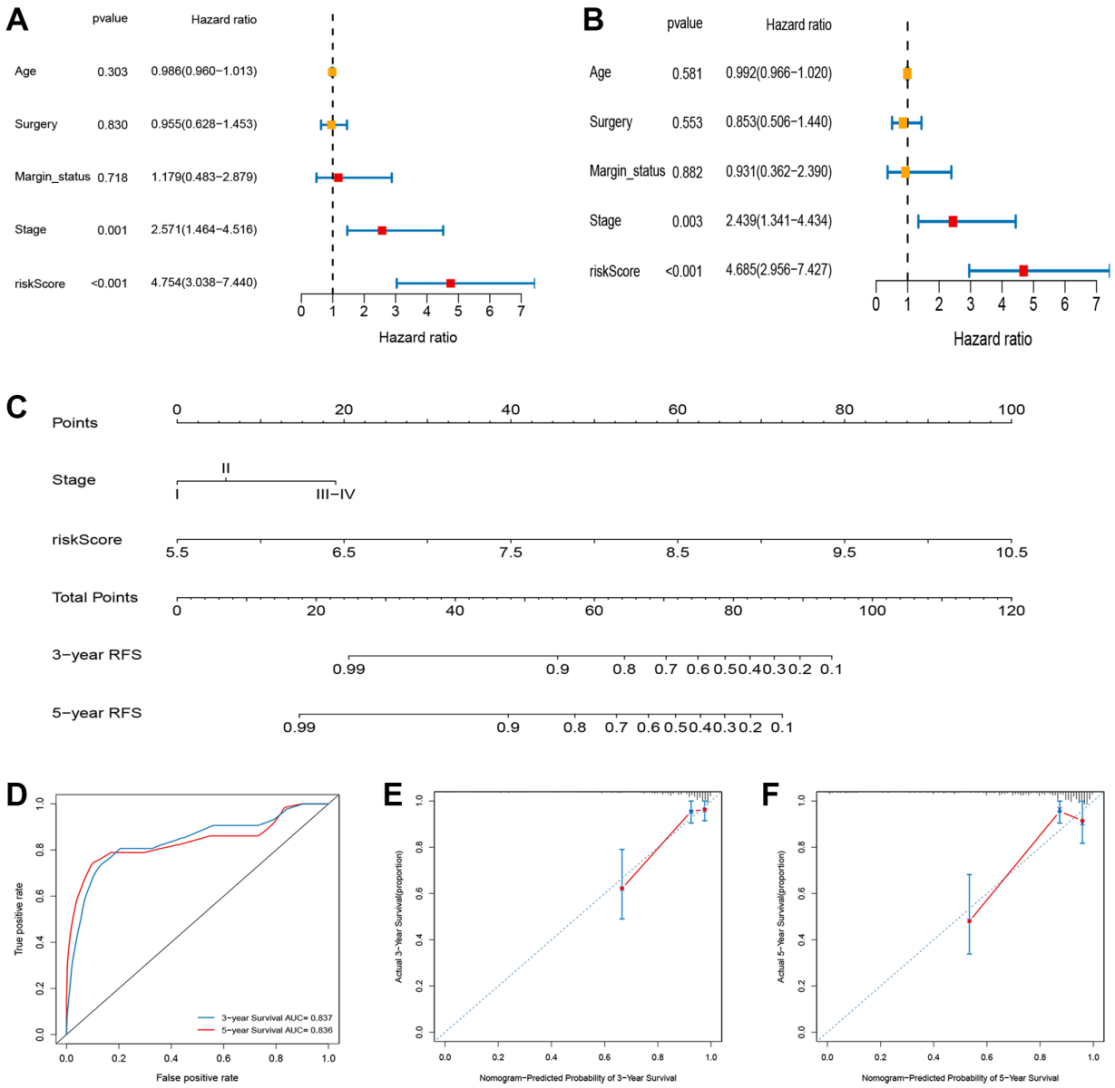
**Construction and evaluation of nomogram for survival prediction of BC patients based on risk score and clinical variables.** (**A**) Univariate Cox regression analysis. (**B**) Multivariate Cox regression analysis. (**C**) Nomogram for predicting the 3- and 5-year RFS of BC patients. (**D**) ROC curves for 3- and 5-year RFS of the nomogram. (**E**, **F**) Calibration curves for predicting 3- and 5-year RFS.

Based on the univariate analysis, the risk score and several clinical features, including age, tumor stage, surgery type, and margin status and were integrated to evaluate their independent prognostic significance in BC. As shown in Figure 9A and 9B, univariate and multivariate analyses revealed a significant correlation between RFS of BC patients, and tumor stage and risk score. To establish a quantitative method for BC prognosis, we used a prognostic nomogram according to tumor stage and risk score (Figure 9C). The AUCs for the 3- and 5- year RFS predictions were 0.837 and 0.836, respectively (Figure 9D). The calibration curve revealed the prediction value of the nomogram and demonstrated high accuracy of the predicted survival (Figure 9E and 9F).

### Functional analysis

**Figure 10:**
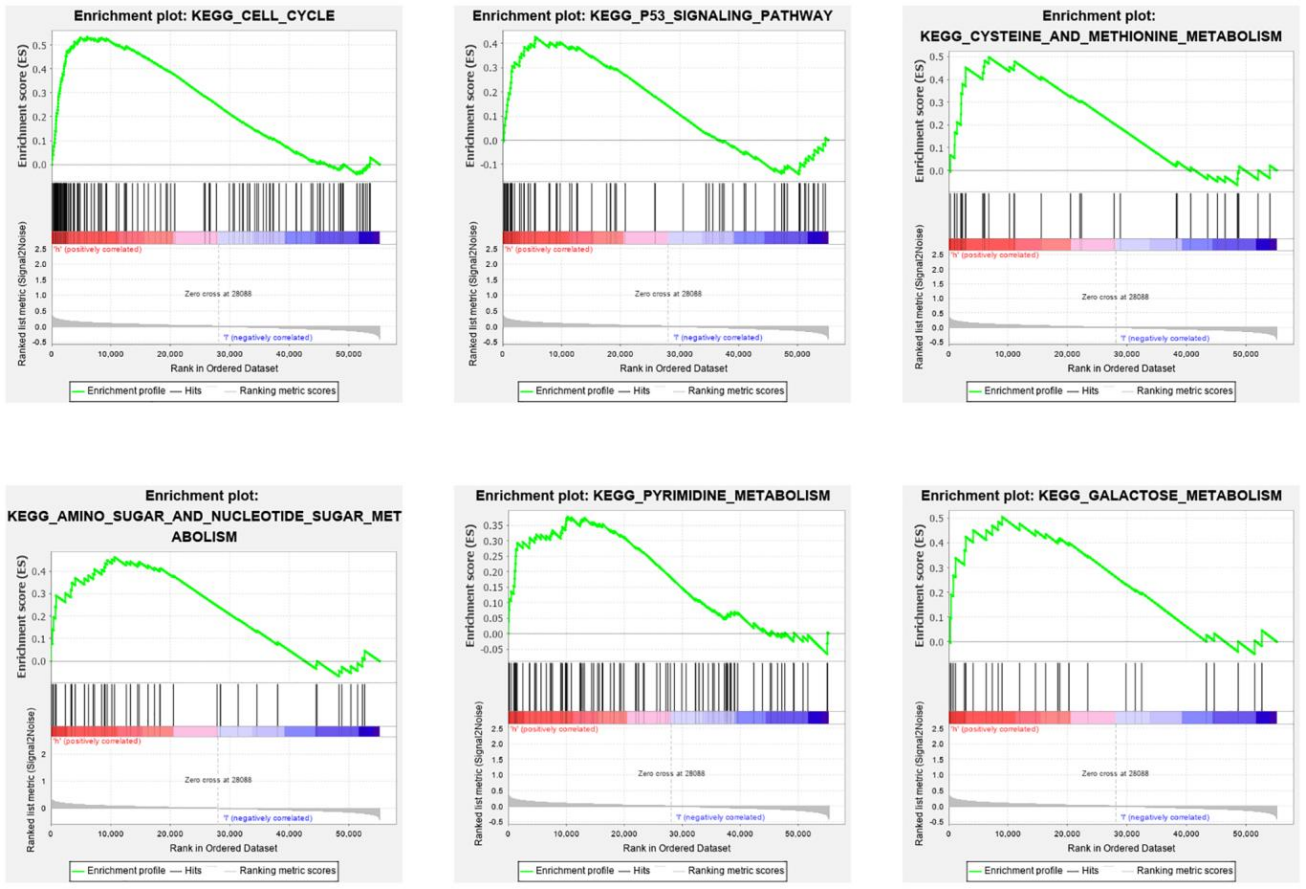
**GSEA analysis of differentially expressed genes in high and low risk groups.** The GSEA analysis revealed a remarkable enrichment of tumor and metabolism-related phenotypes in the high-risk group.

The GSEA was conducted to explore the changes and possible mechanisms in BC patients with different scores. Several signaling pathways were significantly enriched in high- and low-risk group patients (false discovery rate < 0.25 and *P* < 0.05), but there was a different enrichment pattern in the two groups. The cell cycle, P53 pathway, amino sugar, and nucleotide sugar metabolism, pyrimidine metabolism, cysteine, and methionine metabolism, and galactose metabolism pathways were highly gathered in the group of high-risk (Figure 10).

## DISCUSSION

Ferroptosis plays a vital role in maintaining cell survival. Since cancer cells have a greater iron demand than normal cells, they are extremely susceptible to ferroptosis [10]. The therapeutic effect of antineoplastic drugs is far from satisfactory because of the intrinsic and acquired caspase-dependent resistance of cancer cells to apoptosis. Treatments aimed at ferroptosis are promising since they may overcome the deficiencies of traditional apoptosis-inducing chemotherapeutic agents. In addition, a recent study has shown that CD8+ T cells activated by immunotherapy can induce ferroptosis in cancer cells and increase their sensitivity to immunotherapy [20]. Hence, ferroptosis induction may provide a promising therapeutic strategy in cancer treatment, especially in cancers resistant to traditional chemotherapy or immunotherapy. Ferroptosis is closely associated with tumor development and anti-tumor immunity. However, there is still a lack of information about the relationship between BC and ferroptosis.

Our high-throughput “omics” data combined with bioinformatics analysis provided a valid and economical approach to analyze the prognostic value of FRGs in BC. First, we combined the mRNA expression profiles with the survival time and status of patients retrieved from TCGA database, and successfully identified FRGs. A robust FRG-related prognostic signature was established by LASSO regression model for predicting the RFS of patients with breast cancer. We divided patients into two risk groups (low and high) and found statistically significant differences in their survival rates. The tdROC curve and two independent GEO datasets indicated that signature endows a good predictive performance. Analyses of immune infiltrates, TME, immune checkpoints, mutations, drug susceptibility, and clinicopathological features revealed significant differences between the two score groups. The GSEA of the high-score group revealed significant enrichment in cancer-related and metabolism-related processes and pathways. Additionally, a nomogram combining FRG-signature with clinical features was constructed to verify the robustness of the model for speculating RFS in breast cancer patients.

Tumor progression is inextricably linked with the tumor microenvironment, which consists of stromal components, endotheliocytes, mesenchymal stem cells, tumor-associated fibroblasts, and immunocytes [21]. With the recent development of technologies such as RNA-seq, it is possible to systematically analyze the tumor microenvironment and the functional diversity of tumor-infiltrating immune cells that influence and predict the sensitivity of patients to immunotherapy [22]. In this study, we found that the FRG signature significantly correlated with the infiltration levels of immune cells of BC patients. We also assessed the correlation of the signature with the response to immunotherapy. The 30 immune checkpoints, including PD-1, PD-L1, and CTLA-4 was significantly upregulated in the low-risk group, suggesting that immune checkpoint inhibitors could be more effective in BC patients with the low-risk signature score.

Ferroptosis has been reported to participate in the development and progression of BC. Glutathione peroxidase 4 (GPX4) is the main regulator of ferroptosis, which is pivotal for triple-negative BC cell growth. Song et al. [17] found that GPX4 expression was upregulated in gefitinib-resistant cells and knockdown of GPX4 *in vitro* and *in vivo* inhibited cell viability, reduced clonal formation, promoted apoptosis, and increased the cell sensitivity to gefitinib by promoting ferroptosis. Zhang and colleagues [23] recently reported that suppression of circRHOT1 inhibited cell proliferation, invasion and migration, and promoted apoptosis of BC cells. Knockdown of circRHOT1 significantly upregulated the levels of reactive oxygen species (ROS), iron, and Fe^2+^ in BC cells. CircRHOT1 promoted the malignant progression and attenuated ferroptosis of BC cells through the miR-106a-5p/STAT3 axis [26]. Among the 10-FRG signature identified in this study, CHAC1, GCLM, and HMOX1 are closely associated with ferroptosis in BC cells. Chen et al. [24] showed that CHAC1 degradation of GSH enhanced cystine-starvation-induced necroptosis and ferroptosis through the activated GCN2-eIF2α-ATF4 pathway in triple-negative BC cells. Carlisle et al. [25] found that up-regulation of SLC7A11 increased GSH production and inhibited ferroptosis in BC cells. Several studies have indicated that GCLM is a key gene of ferroptosis and its dysregulated expression is significantly associated with the occurrence and development of cancers [26, 27]. HMOX1, also known as HO-1, has been reported to promote curcumin-induced ferroptosis in BC cells [28]. Kato et al. [29] showed that treatment with zinc protoporphyrin 9, which is a specific inhibitor of HO-1, significantly reduced the cell death induced by MI-463 plus auranofin. Yang et al. [30] demonstrated that the ferroptosis sensitivity in ovarian and renal cancers is regulated by cell density through TAZ-ANGPTL4-NOX2 and TAZ-EMP1-NOX4 pathway, respectively.

Our analysis provides new insights into the prognosis and treatment targets of BC. Nevertheless, a few limitations of this study should be taken into consideration. First, the information on BC is incomplete, which may reduce the prediction accuracy of the nomogram, although it is difficult to find a suitable dataset in other databases to validate this nomogram. Second, our study was conducted solely based on data from a public database, and the gene signature was identified mainly with retrospective datasets. Therefore, inherent case selection bias may have influenced the results. In addition, the specific effect and mechanism of the 10 FRGs in CRC remain unclear, and the expression profiles of the 10 FRGs combined with clinical validation in the patients of the prospective cohort need to be proven.

## CONCLUSIONS

In this study, we established a ferroptosis-related prognostic signature that could be used to predict the prognosis and immune responses of BC patients. Our proposed signature can not only be used for clinical stratified management of patients but also lay the foundation stone for future studies investigating ferroptosis as the therapeutic target in BC.

## MATERIALS AND METHODS

### Data collection

The original profiles of mRNA and clinicopathological data of BC cases were obtained from TCGA database, including 1104 tumor samples and 113 normal samples. 179 normal breast tissue data were obtained from the GTEx database by UCSC Xena website (http://xena.ucsc.edu/). After screening, the samples without clinical data were removed. A total of 318 BC patients were included in the analysis. BRCA-related datasets GSE21653 and GSE25066, obtained from the GEO database, were used as validation cohorts. The complete expression profile data and survival information of 241 and 309 BC patients were extracted from the GEO datasets, respectively. In addition, the 267 FRGs were acquired based on the FerrDb database (http://www.zhounan.org/ferrdb/).

### Establishment of ferroptosis-related gene signature

The FRGs strongly related to the RFS were determined to establish an FRG signature for BC using least absolute shrinkage and selection operator (LASSO) Cox regression analysis with “glmnet” and “survival” R package. $\text{Risk core}=\sum_{i=1}^{n}Coef_{i}\times Expr_{i}$, where Coefi is the coefficient and Expri is the expression value of the corresponding FRGs. The risk scores of BC patients were calculated using the risk assessment model. The samples were assigned to low- or high-risk groups, respectively, based on the cutoff values calculated by the “survminer” package in R. Log-rank tests and Kaplan-Meier curves were performed to assess the efficiency of RFS in different risk patients. The predictive ability of the signature was analyzed by ROC curves by “SurvivalROC” R package.

### Validation of the ferroptosis-related gene signature

The strength of the prognostic signature in predicting the survival probability of patients was further verified by GEO datasets (GSE21653 and GSE25066). Using the same method as that used in the training set, the risk score of each patient and the corresponding median risk scores were calculated separately, after which the patients were grouped high-score and low-score. Similarly, the Kaplan-Meier method was employed to assess the efficiency of RFS in low- and high-score groups described above.

### Immune infiltration analysis

The ssGSEA was employed to calculate the infiltration levels of 16 types of immune cells and 13 immune-related pathways in BC [31]. The tumor microenvironment (TME) score of each BC patient was estimated using the ESTIMATE algorithm [32]. In addition, the expression of immune checkpoints was used to examine the molecular relationship with the prognostic signature.

### Comprehensive analysis of the signature

To compare the mutation load between the two score groups, we assessed the association of the signature with the somatic mutation count and tumor mutation burden (TMB). In addition, integrated with the gene expression of TCGA BRCA samples, the algorithm-driven by “pRRophetic” R package based on ridge regression analysis was applied to analyze antineoplastic drug susceptibility in the low- and high-risk groups based on the prognostic model. The half-maximal inhibitory concentration (IC50) was used to assess the antineoplastic drug susceptibility; patients with lower IC50 were more sensitive to antineoplastic drugs.

### Clinical correlation analysis of the prognostic signature

Clinical correlation analysis was performed to evaluate the correlation between risk score and clinical factors, including age, TNM stage, surgery, margin status, and tumor status.

### Building a nomogram

We used Cox regression analysis to select clinical prognostic factors along with risk status to establish a nomogram to foresee the RFS rates for 3 and 5 years of patients. The ROC curve and the calibration plot of the patients’ long-term survival probability (3- and 5-year probability) examined the accuracy and divergence of the nomogram.

### Functional analysis

GSEA based on the KEGG and hallmark gene sets was used to investigate the biological pathways and functions associated with the risk signature. The “Expression datasets” were made and imported as required by the software, and the “gene set database” was selected “c2.cp.kegg.v7.0.symbols.gmt (Curated)”; the number of permutations was set to 1000 times, and the “phenotype labs” were set to high- versus low-risk group. After permutations, a rich gene set was obtained using *P* < 0.05 and false discovery rate (FDR) *q* value < 0.25.

### Availability of data and materials

All samples and files were supported by GEO (https://www.ncbi.nlm.nih.gov/geo) and TCGA database (http://www.cancer.gov/tcga).

## Supplementary Materials

Supplementary Figure 1
