# Supplementary material for: Identification and validation of a ferroptosis-related gene signature predictive of prognosis in breast cancer
Source: Aging (Albany NY). 2021 Sep 9;13(17):21385–99. doi: 10.18632/aging.203472 (PMC8457571; doi:10.18632/aging.203472)
Supplement: Supplementary Figure 1 [file aging-13-203472-s001.pdf]

SUPPLEMENTARY FIGURE

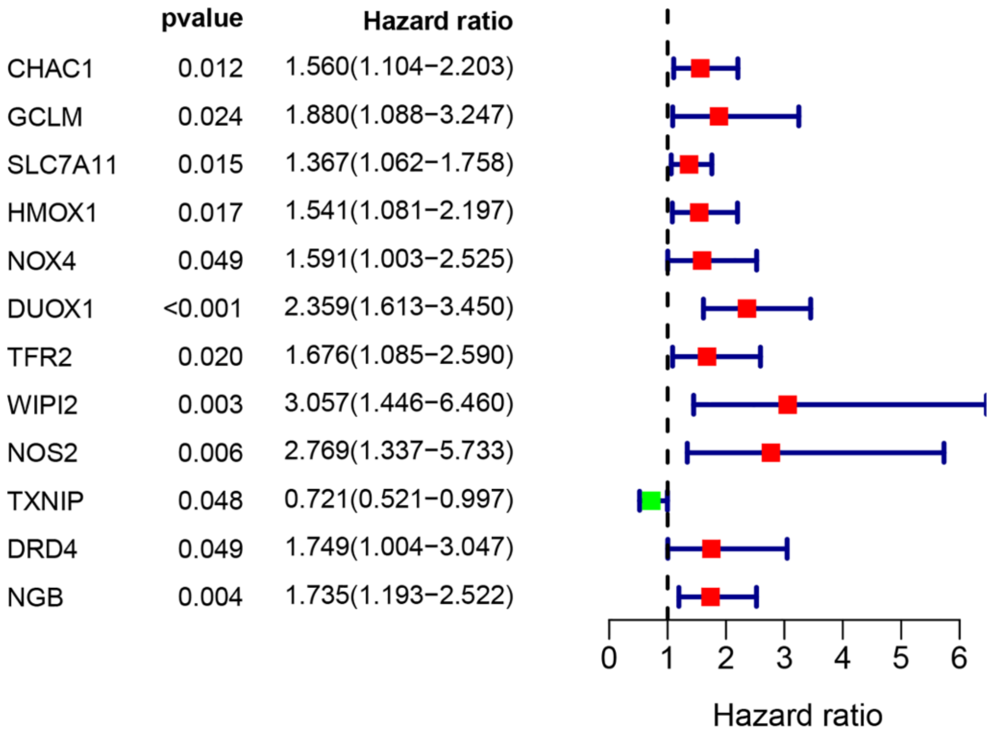

Supplementary Figure 1. Forest plot of 12 candidate prognosis-related FRGs selected by univariate Cox regression analysis.
